# Clinical SARS-CoV-2 Kinetic Profiles Are Dependent on the Viral Strain and Host Vaccination Status

**DOI:** 10.1128/spectrum.04469-22

**Published:** 2022-12-01

**Authors:** Manjula Gunawardana, John M. Cortez, Jessica Breslin, Simon Webster, Nash D. Rochman, Peter A. Anton, Marc M. Baum

**Affiliations:** a Department of Chemistry, Oak Crest Institute of Science, Monrovia, California, USA; b National Center for Biotechnology Information, National Library of Medicine, Bethesda, Maryland, USA; University of Mississippi Medical Center

**Keywords:** SARS-CoV-2 kinetics, proliferation phase, viral dynamics, workplace surveillance clinical study

## Abstract

The severe acute respiratory syndrome coronavirus 2 (SARS-CoV-2) infection kinetics in a real-world, clinical setting represent a knowledge gap in understanding the underlying coronavirus disease 2019 (COVID-19) pathogenesis. There are scant reports of the dynamics describing the two principal components of the viral life cycle, namely, the rapid proliferation and slower clearance phases. Here, we present results from an ongoing workplace clinical surveillance study during which two vaccinated participants became infected with SARS-CoV-2 Omicron variant (BA.1. lineage). The subjects were followed longitudinally with high temporal resolution, allowing the kinetics of both viral phases to be characterized. The viral doubling times in the proliferation phase (3.3 to 3.5 h) and maximum measured viral loads were similar to those observed for unvaccinated individuals infected with an earlier SARS-CoV-2 strain. However, the clearance phase was much shorter in the current study and unexpectedly displayed a multimodal profile. Longitudinal whole-genome SARS-CoV-2 sequencing identified a stable mutation that arose in one of the participants over the 2-week period of positivity. Our small study provides rare insight into the clinical SARS-CoV-2 dynamics, with significance for public health measures and the biology underlying COVID-19.

**IMPORTANCE** We are conducting an ongoing SARS-CoV-2 workplace clinical study based on frequent, longitudinal disease surveillance of staff and household members. Here, we investigated the viral dynamics in two recently vaccinated participants who became infected with the same Omicron variant of SARS-CoV-2. Because the subjects were enrolled in our study, we were able to track the entire viral life cycle with high temporal resolution, with samples collected every 12 h. Surprisingly, the short viral proliferation phase and maximum viral loads in nasal swab samples were similar to our previous observations with unvaccinated participants and an earlier viral strain. However, the decay phase, indicative of viral clearance, was much shorter here. Our results provide a rare, real-world glimpse of the clinical SARS-CoV-2 replication kinetics, potentially impacting immediate therapies and awareness of earlier and greater transmission potential.

## OBSERVATION

A comprehensive understanding of severe acute respiratory syndrome coronavirus 2 (SARS-CoV-2) dynamics throughout the viral growth cycle is key to describing the underlying disease pathogenesis and is needed to inform effective public health measures and clinical management policies. Since 23 March 2020, we have been conducting a continuous, ongoing workplace clinical study involving the longitudinal and intensive characterization of coronavirus disease 2019 (COVID-19) prevalence and incidence (1). This intensely sampled observational study has enabled participants who developed COVID-19 to be identified in the early stages of exponential viral growth (i.e., proliferation phase) and has allowed them to be observed longitudinally via serial measurements with high temporal resolution (2). In our previous study, we followed unvaccinated participants who developed COVID-19 in late 2020 or early 2021, including measurement of viral RNA copy numbers by reverse transcription-quantitative PCR (RT-qPCR) analysis of nasal swab samples. We successfully characterized the viral kinetics of the rapid proliferation and slow clearance phases. The median clinical SARS-CoV-2 doubling time (*t_d_*) during the growth phase was calculated as 3.1 h (range, 2.8 to 5.2 h), and the time between the highest measured viral loads (maximum concentration [*C*_max_]) and a negative test result exceeded 40 days for all participants, extending to 90 days in one case.

**FIG 1:**
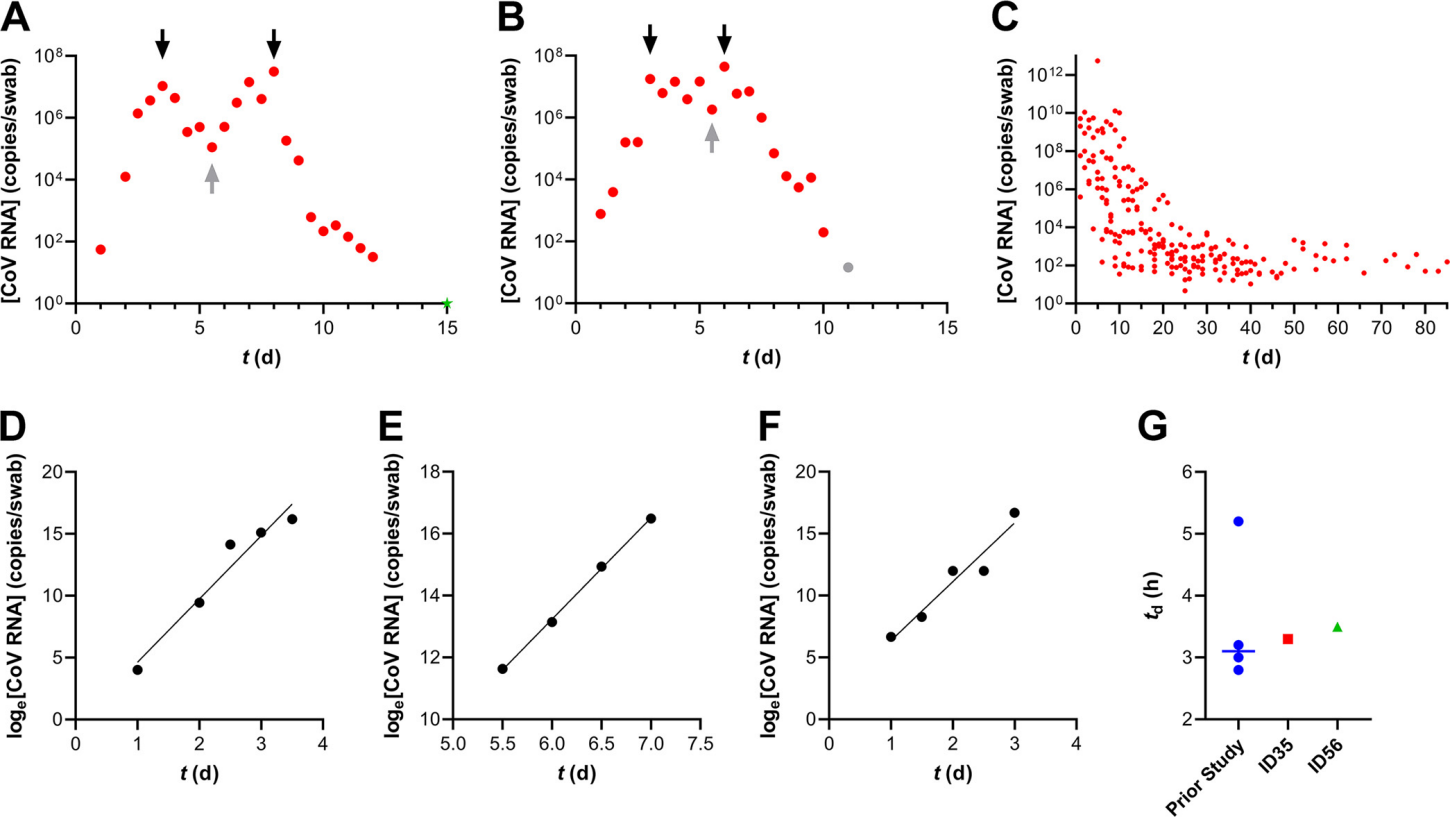
High-resolution SARS-CoV-2 (Omicron BA.1 strain) viral dynamics in two clinical study participants with mild COVID-19. (A and B) The viral load kinetics in nasal swab samples for subject 35 (A) and subject 56 (B) display similar profiles. Red circles, positive; gray circles, inconclusive; green stars, negative. Black arrows identify viral load maxima (*C*_max_), while gray arrows identify viral load minima. (C) Overlaid nasal swab sample viral load trajectories for 8 unvaccinated study participants who became infected with an earlier SARS-CoV-2 strain in our previous study (2). Only positive test results are shown. (D and E) The log-transformed viral loads plotted against time for subject 35 were used to calculate the proliferation phase (*t_d_*, 3.3 h; *R^2^*, 0.947) (D) and midinfection (*t_d_*, 5.1 h; *R^2^*, 0.999) (E) doubling times. (F) The log-transformed viral loads plotted against time for subject 56 were used to calculate the proliferation phase doubling time (*t_d_*, 3.5 h; *R^2^*, 0.934). (G) Comparison of the viral proliferation doubling times from the current study with those in our previous study (2) involving unvaccinated participants infected with an earlier SARS-CoV-2 strain. Participant SARS-CoV-2 vaccination histories were as follows: subject 35, Pfizer-BioNTech (19 March 2021 and 9 April 2021) and Moderna (13 November 2021); subject 56, J&J-Janssen (1 April 2021) and Pfizer-BioNTech (27 October 2021).

Here, we followed two participants, i.e., a male subject (subject 35) and a female subject (subject 56), 58 to 67 years of age, who developed COVID-19 independently between 27 December 2021 (subject 35) and 3 January 2022 (subject 56), the only participants to become infected with SARS-CoV-2 during this period. Both subjects were vaccinated at the time they became infected with SARS-CoV-2 (see the legend to Fig. 1), with no health-concerning conditions, and they were not receiving any immunomodulating medication. They initially developed symptoms within hours of their first positive RT-qPCR test results (subject 35, approximately 4 to 5 h after sample collection; subject 56, approximately 12 to 15 h before sample collection). The last positive SARS-CoV-2 test result for subject 35 was 11 days postdiagnosis, with symptom duration being 6 days. For subject 56, the last positive test result was 10 days postdiagnosis, with resolution of symptoms in 12 days.

Immediately following their first positive results, nasal swab samples were collected every 12 h for viral load analysis by RT-qPCR. At many time points, paired nasal samples were collected for SARS-CoV-2 whole-genome sequencing. The results are summarized in Fig. 1 and capture the complete viral growth cycles.

The *t_d_* and *C*_max_ values were comparable to those observed in our previous study (2) (Fig. 1G), but the clearance phase was much shorter, with both participants being SARS-CoV-2 RNA positive for less than 2 weeks (Fig. 1A and B). The observed rapid clearance could be ascribed to the viral strain, the vaccination status of the participants, or a combination of host-virus factors. It has been suggested that amplification and detection of the short sequences targeted by primers used in the clinical RT-qPCR tests may measure SARS-CoV-2 RNA fragments rather than infectious virus, thereby biasing the length of the observed clearance phase. Our results contradict this rationale, because we previously measured SARS-CoV-2 RNA in COVID-19 patients for up to 90 days (2), while the time from *C*_max_ to undetectable was less than 1 week in the current study using the same methods.

We also measured a nonmonotonic trend in nasal viral loads over time for both participants, an observation that was more pronounced for subject 35 (Fig. 1A). Interestingly, the doubling time in the second phase of viral growth was considerably longer than that in the first (5.1 versus 3.3 h) (Fig. 1D and E).

Paired nasal swab samples from the aforementioned participants also were prepared for SARS-CoV-2 whole-genome sequencing (subject 35, 18 samples; subject 56, 14 samples). Sequence analysis identified the viral strain as Omicron BA.1. The initial viral sequences for the two individuals were nearly identical and could be distinguished by only a single residue, i.e., S R158S, which was observed for subject 56 but not subject 35 and may be relevant to antibody escape (3). Participants had no physical contact and acquired the infection from independent sources. In addition, we analyzed the sequences longitudinally to determine whether any viral mutations emerged in the 14-day window of host-supported viral replication. After accounting for likely sequencing errors, we identified a single mutation that emerged during the period of observation (between 7 a.m. and 7 p.m. on 4 January 2022) and persisted within subject 35. It is notable that 546P is the residue that was conserved among all sequences obtained from subject 56 as well, representing an inherited mutation relative to the ancestral sequence within that subject.

Our results engender a number of caveats. First, only a small study population was observed, predicated by the difficulty of prospectively identifying asymptomatic individuals who just became infected with SARS-CoV-2 and subsequently following those individuals longitudinally with a high sampling frequency. Additional studies are needed to determine to what degree our observations are generalizable. Second, the concept of doubling time is derived from the biology underlying cellular division in microorganisms such as bacteria and does not strictly apply to viruses. However, the parameter is useful in describing the kinetics underlying the exponential expansion of viral populations. Third, viral loads were measured by RT-qPCR amplification targeting specific regions of the SARS-CoV-2 nucleocapsid protein gene transcripts. The measurements may not be fully representative of infectious virus concentrations.

The current report represents a timely and impactful contribution toward elucidating the kinetics of the SARS-CoV-2 Omicron variant (BA.1. lineage) proliferation, with a doubling time of 3.3 to 3.5 h and time to highest viral concentration of <3 days, and documents the clearance time under clinical conditions in middle-aged participants. Our results provide a real-world context for interpreting human challenge study data obtained under controlled conditions using ancestral viral strains and healthy, young adult subjects (4).

### Methods.

**(i) Ethics statement.** All human research under OCIS-05 (Longitudinal Characterization of COVID-19 Prevalence and Incidence in a Small Working Institution with Both Public Health and Diagnostic Aims) was approved by the Aspire institutional review board (IRB) (Aspire study number 1281548) and conducted according to the Declaration of Helsinki. All study participants provided written informed consent or assent.

**(ii) Clinical study design.** The workplace SARS-CoV-2 surveillance clinical study was initiated by the Oak Crest Institute of Science (https://www.oak-crest.org), a small nonprofit academic science research organization located in Monrovia, California, on 23 March 2020, has been running without interruptions, and is ongoing at the time of writing. The study design has been described in detail elsewhere (1, 2). No changes in the testing strategy or methods other than increasing the testing frequency to once every 12 h during the period of SARS-CoV-2 positivity were adopted here.

**(iii) Calculation of SARS-CoV-2 doubling time.** The *in vivo* SARS-CoV-2 doubling time (*t_d_*) during the exponential growth phase (i.e., proliferation phase) was calculated according to methods described elsewhere (2).

**(iv) Whole-genome SARS-CoV-2 sequencing.** Nasal swab samples for sequencing were preserved in RNA shield buffer (300 μL, R1200-125; Zymo Research, Irvine, CA), frozen at −80°C, and stored and transported at −80°C. Samples were prepared for whole-genome SARS-CoV-2 sequencing using the tailed amplicon method (5) at the University of Minnesota Genomics Center (UMGC) (Minneapolis, MN). Briefly, RNA was extracted using the QIAamp viral RNA minikit (52904; Qiagen, Germantown, MD), and cDNA and amplicon libraries were generated according to the published ARTIC v3 protocol (5). The sequencing method employed 95 PCR primer pairs to tile the SARS-CoV-2 genome with overlapping amplicons in a total of four multiplexed amplification reactions, to achieve virtually complete genomic assembly.

**(v) SARS-CoV-2 sequence analysis.** Genomes sampled across multiple time points for two individuals were aligned to the reference sequence Wuhan-Hu-1 (GenBank accession number NC_045512.2) using MAFFT (6). All gaps were adjusted to respect the open reading frames (ORFs) of the reference sequence as described previously (7). Only one substitution in a noncoding region that does not precede ORF1ab or follow ORF10 (A28271T, conserved in all samples) was observed, and noncoding regions were removed. Only one conserved insertion, consistent with S 214 insEPE (8), was identified in both individuals, as well as several nonsynonymous substitutions that enabled the identification of all variants as Omicron BA.1. The genomes from the two individuals could be distinguished by a single residue, S R158S, observed for subject 56 but not subject 35 across all samples for each individual. Additionally, several substitutions and small deletions that were not temporally conserved and were attributed to sequencing errors were observed (S11 GTC→GTT, S del151-2, S H954Q, S K969N, S F981L, E I9T, and 7b T40I).

**(vi) Data analysis.** Data sets were analyzed using GraphPad Prism version 9.4.0 (GraphPad Software, Inc., La Jolla, CA).

**(vii) Data availability.** SARS-CoV-2 nucleotide sequences have been deposited in GenBank (accession numbers OP700058-OP700088). All other data supporting the findings of this manuscript are available from the corresponding author upon reasonable request.
